# Recording and Simulating Proton-Related Metabolism in Bacterial Cell Suspensions

**DOI:** 10.3389/fmicb.2021.654065

**Published:** 2021-04-29

**Authors:** Heribert Cypionka, Jan-Ole Reese

**Affiliations:** Institute for Chemistry and Biology of the Marine Environment, Carl-von-Ossietzky University of Oldenburg, Oldenburg, Germany

**Keywords:** vectorial proton translocation, dissimilatory sulfate reduction, dissimilatory nitrate reduction to ammonia, ATP synthase activity, Desulfovibrio desulfuricans, Michaelis-Menten kinetics, proton-sulfate symport

## Abstract

Proton release and uptake induced by metabolic activities were measured in non-buffered cell suspensions by means of a pH electrode. Recorded data were used for simulating substrate turnover rates by means of a new freeware app (*proton.exe*). The program applies Michaelis-Menten or first-order kinetics to the metabolic processes and allows for parametrization of simultaneously ongoing processes. The simulation includes changes of the transmembrane ΔpH, membrane potential and ATP gains, and demonstrates the principles of chemiosmotic energy conservation. In our experiments, the versatile sulfate-reducing bacterium *Desulfovibrio desulfuricans* CSN (DSM 9104) was used as model organism. We analysed sulfate uptake by proton-sulfate symport, scalar alkalinization by sulfate reduction to sulfide, as well as nitrate and nitrite reduction to ammonia, and electron transport-coupled proton translocation. Two types of experiments were performed: In oxidant pulse experiments, cells were kept under H_2_, and micromolar amounts of sulfate, nitrate or nitrite were added. For reductant pulse experiments, small amounts of H_2_-saturated KCl were added to cells incubated under N_2_ with an excess of one of the above-mentioned electron acceptors. To study electron-transport driven proton translocation, the membrane potential was neutralized by addition of KSCN (100 mM). H^+^/e^–^ ratios of electron-transport driven proton translocation were calculated by simulation with *proton.exe*. This method gave lower but more realistic values than logarithmic extrapolation. We could verify the kinetic simulation parameters found with *proton.exe* using series of increasing additions of the reactants. Our approach allows for studying a broad variety of proton-related metabolic activities at micromolar concentrations and time scales of seconds to minutes.

## Introduction

All living cells, mitochondria, chloroplasts or bacteria cause various types of pH changes. First, production or consumption of acidic or basic metabolites results in permanent pH effects. As such reactions change the amount of protons in the system they are named scalar. By far more common are vectorial processes which translocate protons or metabolites across a membrane without changing their overall amount. The resulting pH effects are transient and compensated by counteracting cyclic transport processes. Some vectorial processes – named electroneutral – are not going along with a net charge transfer (e.g., transport of an anion A^–^ in symport with a single proton H^+^). By contrast, electrogenic transport (e.g., symport of 2 H^+^ per anion A^–^) affects the electrical potential across the membrane. The most important electrogenic vectorial processes are proton translocation during respiration and photosynthesis, which build up the proton-motive force (pmf, composed of the trans-membrane proton gradient, and membrane potential). The pmf is then used to drive ATP conservation by the membrane-bound ATP synthase (or simply ATPase), which is also an electrogenic proton transport system. As the ATPase takes up 3–4 H^+^ per ATP (Cross and Müller, 2004; Steigmüller et al., 2008; Petersen et al., 2012), which before have been pumped out driven by respiratory or photosynthetic electron transport, proton translocation across a membrane is the most common process in living cells. For example, a bacterium respiring a single molecule of glucose with oxygen gains about 30 ATP (Rich, 2003). This involves vectorial translocation of up to 120 protons by the respiratory chain, which will be taken up again by the ATPase. Thus, up to 240 protons per glucose are translocated across the membrane.

Scalar consumption or production of protons can easily by recorded by means of a pH electrode. Vectorial proton transport, however, often remains invisible as the cyclic compensation processes are rather fast. Particularly, electrogenic protons are drawn back immediately by the membrane potential. Therefore one has to neutralize the membrane potential for measurements of electrogenic proton translocation. For this purpose, potassium thiocyanate (KSCN) can be used (Mitchell and Moyle, 1967, 1968). The chaotropic SCN^–^ ion is known to be membrane permeant, thus compensating electrogenic processes and slowing down vectorial proton backflow.

**FIGURE 1:**
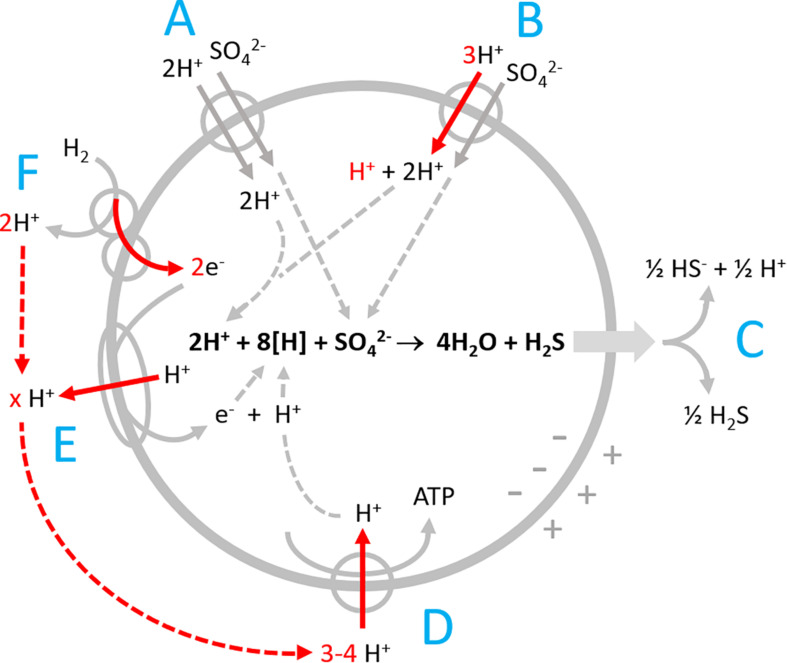
Proton-related processes in *Desulfovibrio* during sulfate reduction with hydrogen. **(A)** Electroneutral proton-sulfate symport; **(B)** electrogenic proton-sulfate symport; **(C)** release of H_2_S by diffusion and partial dissociation to H^+^ + HS^–^; **(D)** ATP synthase; **(E)** proton-translocating electron transport chain; and **(F)** periplasmic hydrogenase coupled to cytochrome c that channels electrons into the cell by vectorial electron transport. Red arrows point to electrogenic vectorial processes.

In our study we analysed H_2_ oxidation by the versatile sulfate reducer *Desulfovibrio desulfuricans*, that is able to utilize nitrate, nitrite, or sulfate as electron acceptor (Steenkamp and Peck, 1981; Seitz and Cypionka, 1986; Dilling and Cypionka, 1990). Both sulfate and nitrate reduction cause scalar alkalinization of the outer bulk medium. While nitrate reduction presumably proceeds in the periplasm (Marietou, 2016), sulfate reduction is known to proceed intracellularly. We have to consider several proton-related transport processes and scalar pH changes (Figure 1).

*Desulfovibrio* species possess periplasmic hydrogenases which allow for generating proton-motive force by a special mechanism named vectorial electron transport (Figure 1F, Badziong and Thauer, 1980; Marietou et al., 2005; Duarte et al., 2018). The 2 H^+^ produced by H_2_ oxidation remain in the periplasm and in equilibrium with the bulk medium until they are taken up via the ATPase (Figure 1D). The electrons derived from H_2_ are channeled electrogenically into the cell via cytochrome c. The eight reducing equivalents (H^+^ + e^–^ or [H]) consumed during sulfate reduction are thus entering the cell via electrogenic processes (Figure 1). Additionally, sulfate reducers have proton-translocating electron transport chains (Figure 1E) allowing for translocation of more than 1 H^+^/e^–^ (the maximum gain possible with vectorial electron transport).

The cells take up sulfate in symport with protons (Cypionka, 1987, 1989, 1994; Marietou et al., 2018). During growth with excess sulfate, the symport is electroneutral (Figure 1A), while sulfate limitation induces electrogenic symport, which is faster and allows for 1000-fold intracellular sulfate accumulation (Cypionka, 1995, Figure 1B). Sulfate reduction forms cytoplasmic HS^–^ and H_2_S. Unlike HS^–^, H_2_S is an uncharged small molecule, and membrane permeant. It leaves the cell by diffusion (Figure 1C) and thereby exports two bound protons, just the amount taken up during electroneutral symport with sulfate. Sulfate reduction will therefore not affect the intracellular proton balance and pH. Outside, H_2_S dissociates partially to 0.5 HS^–^ + 0.5 H^+^ causing a slight acidification. The scalar alkalinization of the bulk medium of 1.5 H^+^ per reduced sulfate is thus brought about by loss of 2 H^+^ during the initial proton-sulfate symport and a compensation of 0.5 H^+^ when the released H_2_S partially dissociates in the bulk medium.

Sulfate transport and scalar alkalinization by sulfide production run simultaneously with the different electrogenic processes described in Figures 1D–F. To visualize and separately assess the electrogenic processes, we performed experiments with a neutralized membrane potential, and we added pulses of either electron acceptor or donor to non-buffered cell suspensions.

In order to simulate the simultaneously ongoing processes, we developed a freeware app (*proton.exe*) describing the metabolic activities by Michaelis-Menten kinetics or – for the ATPase rate – first order kinetics. With its help one can model the ATP gain and changes of the proton-motive force as well. The simulation is applicable to physiological studies with all bacteria that perform proton-related metabolism, could also be used with mitochondria, phototrophic bacteria or chloroplasts, and demonstrates chemiosmotic energy conservation in general.

## Materials and Methods

### Organism and Cultivation

Experiments were carried out with *Desulfovibrio desulfuricans* strain CSN (DSM 9104), grown in a pH-controlled chemostat as described by Cypionka (1986), but without sulfide recording. The reservoir medium was the freshwater mineral medium described by Cypionka and Pfennig (1986), containing lactate (30 mM) and either sulfate or nitrate as an electron acceptor in limiting concentrations (10 mM). The culture was gassed with N_2_. CO_2_ was additionally supplied by the pH regulator set to maintain a pH of 7.0.

### Preparation of Cell Suspensions

The chemostat outflow was collected on ice under N_2_ atmosphere. Cells were harvested by centrifugation (9.950×*g*, 20 min, 4°C, Beckman JA-10), washed once and resuspended in N_2_-saturated 150 mM KCl as described by Kirchhoff et al., 2018.

### Set-Up for Physiological Experiments

Washed cells were diluted with KCl (150 mM) to obtain an OD_436_ (Biochrom Libra S12 UV/Vis Spectrophotometer) between 5 and 40 and a final volume of 2 ml. Experiments were carried out at 30°C in a reaction chamber equipped with a pH electrode (Inlab Micro, Mettler Toledo) and magnetic stirring. Anoxic conditions were obtained by sealing the chamber with a rubber stopper and flushing it with either H_2_ or N_2_ by fine cannulas for 10 min. After the suspension was filled into the chamber, it was bubbled with the respective gas and cells were allowed to equilibrate for 30 min. After this pre-incubation, only the headspace was flushed with gas. Proton translocation was measured using the method described in former studies (Cypionka et al., 1984; Fitz and Cypionka, 1989; Kirchhoff et al., 2018). The membrane potential of the cells was neutralized by addition of 100 mM KSCN. As described above, the membrane-permeable SCN^–^ anions are known to distribute over both sides of the membrane and compensates any electrogenic solute transport by migrating across the membrane. Two types of experiments were performed: In oxidant pulse experiments, cells were incubated under H_2_ to provide an excess of the electron donor. Then micromolar pulses of various electron acceptors (Na_2_SO_4_, NaNO_3_, and NaNO_2_) were added from 1 mM N_2_-flushed stock solutions. For reductant pulse experiments, the cell suspensions were preincubated with either Na_2_SO_4_ or NaNO_3_ (10 mM) under N_2_. Subsequently, H_2_-saturated KCl (150 mM, assumed to contain 680 μM H_2_ at 25°C, Crozier and Yamamoto, 1974) was used to add reductant pulses.

N_2_-flushed 1 mM HCl and KOH (in 150 mM KCl) were added to calibrate the pH measurement and to titrate the suspension back to a neutral pH. pH changes were recorded by an AD converter (ADC-16, Pico Technology).

### Short Description of *proton.exe*

pH recordings were simulated by means of *proton.exe.* This freeware app (Cypionka, 2021, see Supplementary Material for download and tutorial videos) allows for modeling of metabolic processes coupled to chemiosmotic energy conservation in bacterial cells, and generates simulated pH curves resulting from metabolic processes. A freeware app (Glass2k.exe) was used to get semi-transparent windows allowing for overlays of measured and simulated pH curves. For fitting the results with real pH data, one enters technical calibration parameters of the assay like buffering capacity, sensitivity, and response time of the pH electrode. Some biological parameters describe the cell suspension, e.g., cell number and size, membrane potential, number of protons translocated per electron by the electron transport system, as well as the numbers of protons taken up per ATP, and the ATPase rate. If some of these data are not known one can start with arbitrary numbers and learn how those change the simulated pH curves. *proton.exe* can be used without real data. Furthermore, it calculates ATP gains and changes of the proton-motive force. The metabolic reactions are started by (virtual or real) additions of micromolar pulses of electron donors or acceptors.

Uptake and metabolic rates (*v*) are assumed to depend on the substrate concentration [S] and calculated once per second assuming Michaelis-Menten kinetics according to:

$$V=\left(V_{\max}\cdot \left[\text{S}\right]\right)\,/\,\left(K_{\text{M}}+\left[\text{S}\right]\right)$$

with an estimated maximum rate *V*_max_, and Michaelis constant *K*_M_.

For proton uptake rates by the membrane ATPase first-order kinetics is assumed, i.e., the dependence on the proton gradient across the membrane (ΔpH, difference to the start value ΔpH_0_, which is assumed to be in equilibrium):

$${\text{H}}^{+}\,\text{uptake}=A\cdot \left(\Delta \,\text{pH}-\Delta \,{\text{pH}}_{0}\right)$$

with *A* as the ATPase H^+^ uptake rate per sec.

The simulation not only generates virtual pH curves, but also calculates the corresponding concentration changes of reactants and products as well as the ATP gain. The components of the proton-motive force, i.e., ΔpH and membrane potential are calculated as well. The calculated membrane potential, however, is not used as driving parameter for the simulation as the membrane potential in our proton-translocation experiments was neutralized by KSCN. *proton.exe* shows the concentration changes over time graphically and saves the parameters used together with the simulation results in the csv format that can be read by any text editor or spread-sheet program.

### Assessment of the Simulation Quality

All experiments were repeated at least four times. Standard deviations between measured and simulated data were determined with Excel by pairwise comparison. The simulations reached similarities to the original data curves of 86–97% (Supplementary Table 1), with the deviations mainly arising from scattering of the original data. Lower similarities were obtained for calibration pulses, which can be explained by temporary local concentration peaks, generated when HCl pulses were added close to the pH electrode sensor tip.

To check the relevance of the simulation parameters, we did series of experiments changing only the amounts of added oxidant or reductant. By this approach, the concentrations of the metabolites were changed and gave corresponding reaction kinetics. Simulation was performed the same way with all parameters but the added amounts kept constant. As both Michaelis-Menten and first order kinetics describe concentration-dependent reaction rates the accuracy of our simulations proved that we were applying realistic values.

## Results

### Scalar pH Changes Caused by Sulfate and Nitrate Reduction

**FIGURE 2:**
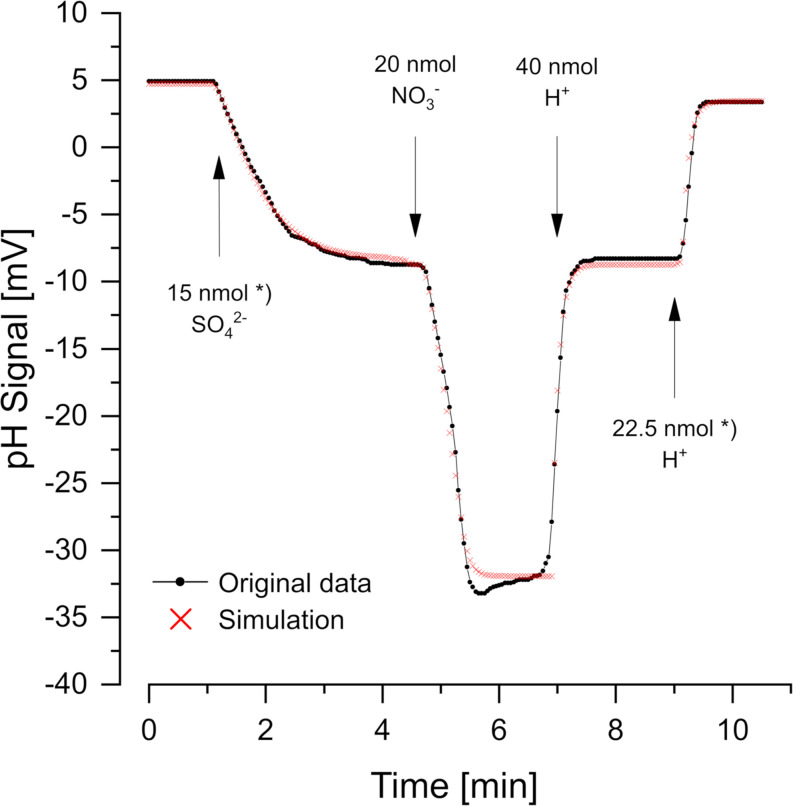
Scalar pH changes correlated with sulfate and nitrate reduction by *Desulfovibrio desulfuricans* CSN. A cell suspension with OD_436_ = 1 was incubated at 30°C in H_2_-saturated 150 mM KCl, before small pulses of 1 mM Na_2_SO_4_, NaNO_3_, or HCl (in 150 mM N_2_-flushed KCl) were added. The red dots show the simulation obtained with *proton.exe* (parameters see Supplementary Material, standard deviations see Supplementary Table 1). *Data from Krekeler and Cypionka, 1995. Note that a satisfying simulation was obtained only if additions of 15 nmol sulfate and 22.5 nmol H^+^ (second calibration pulse) were assumed, while the original publication says 20 nmol sulfate and 30 nmol H^+^. Even without simulation by *proton exe*, it is obvious that the second H^+^ pulse gave a far smaller response than the 30/40 of the first one of 40 nmol H^+^. Further sulfate and H^+^ additions in the original experiment (Krekeler and Cypionka, 1995) confirmed the scalar consumption of 1.5 H^+^ per reduced sulfate.

Figure 2 (data from Krekeler and Cypionka, 1995) shows a cell suspension of *Desulfovibrio desulfuricans* reducing additions of sulfate and nitrate with H_2_ as electron donor. Nitrate was reduced more rapidly than sulfate. The reactions consume 1.5 H^+^ per sulfate and 2 H^+^ per nitrate according to the Eqs 1, 2:

(1) $$4\,H_{2}+{\text{SO}}_{4}^{2-}+1.5\,{\text{H}}^{+}\to 0.5\,{\mathrm{HS}}^{-}+0.5\,H_{2}\,\text{S}+4\,H_{2}\,\text{O}$$

(2) $$4\,H_{2}+{\text{NO}}_{3}^{-}+2\,{\text{H}}^{+}\to 3\,H_{2}\,\text{O}+{\text{NH}}_{4}^{+}$$

To obtain the correct scalar pH effects, the equations are written for pH = 7 and regard the pK values of H_2_S/HS^–^ (7.0) and NH_4_^+^/NH_3_ (9.3). Proton quantification was done by adding calibration pulses of a few nanomoles H^+^ (as 1 mM HCl in 150 mM N_2_-saturated KCl), which also helped to keep the pH of the non-buffered suspension constant. Although not essential for the simple curves in Figure 2, we used *proton.exe* to model the reaction kinetics. The scalar pH effects of sulfate and nitrate reduction were compensated by H^+^ additions according to the Eqs 1, 2 (see Supplementary Material for simulation data files and tutorial #3). However, the simulation led to the discovery of an error in the originally published figure. Both, sulfate reduction and the second H^+^ calibration pulse could only be simulated correctly if 25% smaller additions than originally published were assumed (see legend of Figure 2). When we had published this experiment (Krekeler and Cypionka, 1995) the discrepancy had been overlooked.

### Transient Vectorial pH Changes Related to Proton-Sulfate Symport

**FIGURE 3:**
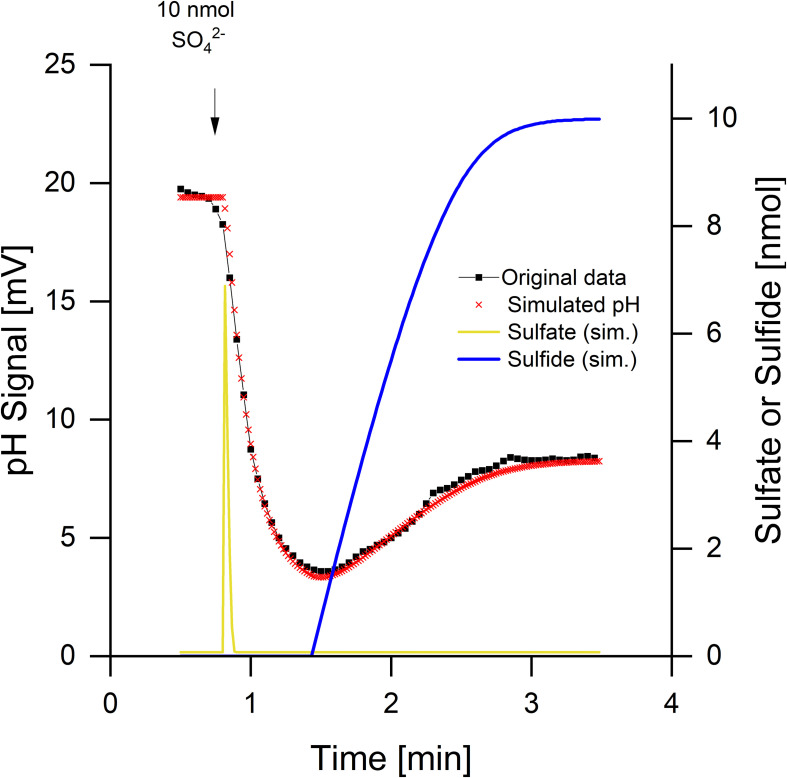
Sulfate reduction by *Desulfovibrio desulfuricans* CSN grown under sulfate limitation. Conditions as described in Figure 1, data from Cypionka (1995). Additionally to the pH curve, sulfate uptake and sulfide release were simulated by means of *proton.exe* (parameters see Supplementary Material, standard deviations see Supplementary Table 1). The simulation includes initial proton-sulfate symport followed by sulfate reduction and release of H_2_S part of which dissociating to H^+^ + HS^–^.

The pH curves during sulfate reduction did not always look like the one in Figure 2. Depending on the physiological state of the cells, a rapid and transient initial alkalinization occurred (Figure 3). Sulfate limitation is known to induce high-affinity sulfate uptake systems which accumulate sulfate in symport with protons (Cypionka, 1987, 1989, 1994). Furthermore, starved cells often do not reduce added sulfate immediately, probably because they have a low internal level of ATP, which is required for sulfate activation. The initial disappearance of two protons can be explained by rapid sulfate uptake via symport with protons. Intracellular sulfate reduction started with a delay and then caused a slight acidification, when H_2_S was diffusing through the membrane and outside partially dissociated to 0.5 HS^–^ + 0.5 H^+^ (Figure 1C). The high-accumulating sulfate transport is electrogenic and probably takes up 3 H^+^ per sulfate (Cypionka, 1989, 1994). The electrogenic third proton, however, will be invisible as long as the cells have their normal membrane potential that will rapidly compensate electrogenic proton transitions (see section “Discussion” and tutorial videos).

Kinetics of sulfate uptake and subsequent reduction could be modeled by *proton.exe*. An accurate simulation (Figure 3, see Supplementary Material for the parameters used) was only obtained if it was assumed that sulfate reduction started after completion of sulfate uptake. Sulfate uptake not coupled to immediate reduction has been reported already by Cypionka (1987; 1989; 1994). The delayed sulfide production was verified by means of a sulfide electrode, when this experiment was published first (Cypionka, 1995). The corresponding bulk sulfate and sulfide concentrations were calculated by *proton.exe* as well (Figure 3).

### Vectorial Proton Translocation Driven by Electron Transport

**FIGURE 4:**
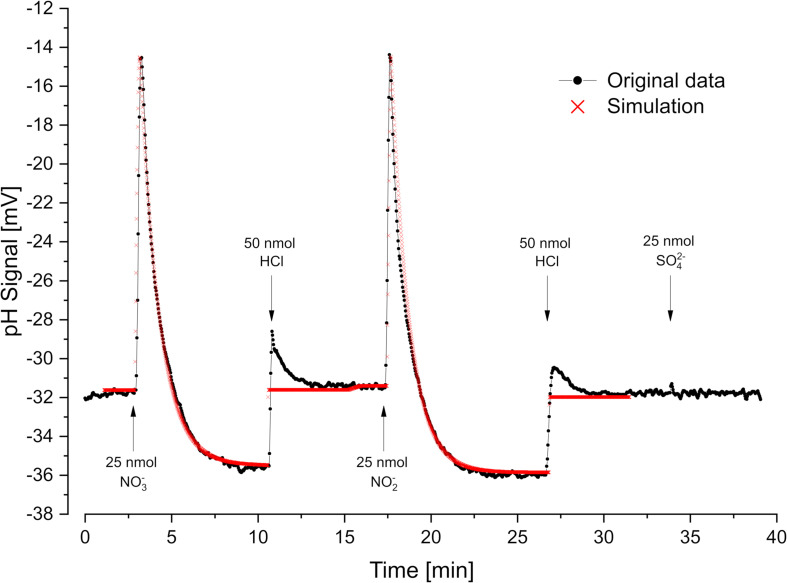
Oxidant pulse experiment. Proton translocation coupled to nitrate- and nitrite reduction. Nitrate-grown cells were incubated under H_2_ in the presence of KSCN (100 mM). Sulfate was not metabolized under these conditions. pH curves and proton translocation were simulated by means of *proton.exe* (parameters see Supplementary Material, standard deviations see Supplementary Table 1). The H^+^/e^–^ ratio was additionally calculated by logarithmic extrapolation back to the time of electron acceptor addition (see Supplementary Figure 1).

In order to study electron-transport driven vectorial proton translocation, the membrane potential of the cells was neutralized by preincubation with KSCN, which did not change the overall reaction rates and final scalar pH changes. However, additional peaks indicating electron-transport driven proton translocation became visible (Figure 4). As expected, these peaks were transient as the protons simultaneously flow back via the membrane ATPase. The full amount of translocated protons is therefore not detectable by a pH electrode.

Proton uptake by the membrane ATPase is assumed to follow first order kinetics (Mitchell and Moyle, 1967, 1968), i.e., it should be proportional to the proton gradient across the membrane. Normally, one calculates the number of translocated protons by exponential extrapolation from the final phase, when there is no more electron transport but still ATPase activity going on. For our experiments, we used *proton.exe* to simulate these processes applying *K*_M_ and *V*_max_ values to transport and metabolic processes. Simulation gave smaller H^+^/e^–^ values than extrapolation, which, however, are more realistic (see section “Discussion”).

### Vectorial Proton Translocation in Oxidant Pulse Experiments

Two types of experiments were performed to study electron transport-driven proton translocation with sulfate and nitrate. In oxidant pulse experiments washed cells were incubated with KSCN under H_2_, and micromolar pulses of sulfate, nitrate or nitrite were added.

Cells grown with nitrate as electron acceptor performed rapid proton translocation with both nitrate and nitrite (Figure 4). The pH curves for nitrate and nitrite reduction looked almost identical which indicated that nitrate reduction to nitrite

(3) $${\text{H}}_{2}+{\text{NO}}_{3}^{-}\to {\text{H}}_{2}\,\text{O}+{\text{NO}}_{2}^{-}$$

is not coupled to proton translocation as already reported by Steenkamp and Peck (1981). Small sulfate pulses added to nitrate-grown cells were not reduced and did not result in proton translocation with nitrate-grown cells (Figure 4). The scalar proton consumption during nitrite reduction followed Eq. 4:

(4) $$3\,H_{2}+{\text{NO}}_{2}^{-}+2\,{\text{H}}^{+}\to 2\,H_{2}\,\text{O}+{\text{NH}}_{4}^{+}$$

Simulation of the experiment with *proton.exe* was done based on the model in Figure 1, assuming periplasmic nitrate- and nitrite reduction (Marietou et al., 2005). We obtained a simulated vectorial proton translocation of 12.2 H^+^ per nitrate or nitrite, while the extrapolation method gave a value of 14.4 H^+^. The H^+^/H_2_ ratio was lower with nitrate (3 H^+^/H_2_) than with nitrite (4 H^+^/H_2_) as the nitrate reductase consumes H_2_ without coupling to proton translocation.

### Reductant Pulse Experiments With H_2_

**FIGURE 5:**
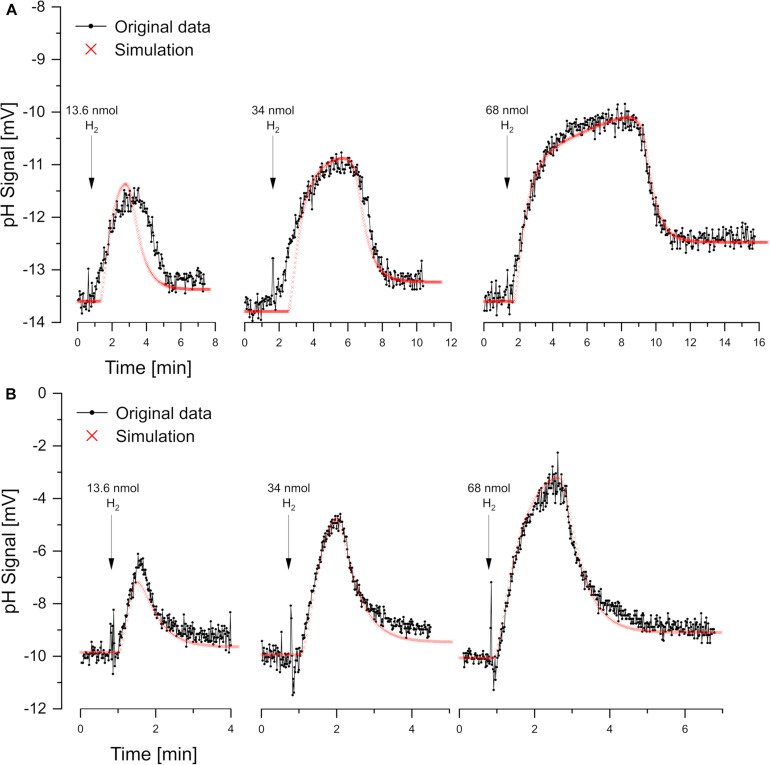
Reductant pulse experiments. Proton translocation induced by H_2_ pulses to cell suspensions incubated in N_2_-saturated KCl with KSCN (100 mM) and **(A)** sulfate-grown cells with 10 mM Na_2_SO_4_ or **(B)** nitrate-grown cells with 10 mM NaNO_3_. Three different H_2_ concentrations were added for each electron acceptor. Correspondingly the simulations by *proton.exe* differ only by the amounts of electron donor added (parameters see Supplementary Material, standard deviations see Supplementary Table 1).

For reductant pulse experiments, the cells were incubated under N_2_ with KSCN and with excess sulfate or nitrate. Small pulses of H_2_ (added as H_2_-saturated KCl) caused vectorial proton translocation. However, the reactions were slower than in oxidant pulse experiment and, depending on the added amount of H_2_, steady-state phase of proton translocation and uptake developed (Figure 5). There was no pH effect that could be attributed to sulfate uptake, which obviously had been accumulated sufficiently. Furthermore, there was no scalar alkalinization as described by the Eqs 1, 2, 4. By contrast, with both sulfate and nitrate as electron acceptor, a slight acidification was observed as already reported by Fitz and Cypionka (1989). The pH curves could be simulated by *proton.exe*. Experiments with three different H_2_ additions confirmed the simulation quality, when the only simulation parameter changed was the amount of electron donor added (Figure 4).

For experiments with steady-state phases, electron-transport-driven proton translocation cannot be calculated by the logarithmic extrapolation method (see section “Discussion”). With *proton.exe*, however, we could simulate the experiments applying the same proton translocation of 1 H^+^/e^–^ (or 2 H^+^/H_2_) for both sulfate and nitrate as electron acceptor. This result is in accordance with the assumption that H_2_ is oxidized by a periplasmic hydrogenase which channels two electrons per H_2_ into the cytoplasm via cytochrome c (Marietou et al., 2005), while the two protons resulting from H_2_ oxidation remain extracellular (Figure 1F). This so-called vectorial electron transport (Badziong and Thauer, 1980; Duarte et al., 2018) generates a proton-motive force without requiring proton pumps.

## Discussion

### pH Recording and Simulation as a Powerful Tool

Acid-base titration is a well-known quantitative method, and pH electrodes are rather sensitive tools. For example, changing the pH by one unit from 7 to 8 (i.e., 10^–7^–10^–8^ mol H^+^/l) in a non-buffered assay, corresponds to a H^+^ concentration decrease by 90 nM (giving a signal response of −59 mV). The high sensitivity allows for physiological experiments at substrate limitation and natural concentration levels. We demonstrated how to measure the reduction of sulfate and nitrate (Figure 2) and nitrate and nitrite (Figure 4) in the same assay within a few minutes. As many metabolic reactions cause scalar H^+^ consumption or release, there are many more physiological activities that can be recorded by our approach.

Simulation of the pH curves as done in our study is not required if only scalar pH effects are visible. However, as shown in Figure 2 the simulation lead to the detection and correction of a discrepancy in published data. Although there are several parameters adjustable in *proton.exe*, the calibration pulses and variations of the additions restrict the simulations and lead to reproducible results.

### Vectorial Proton Translocation: Extrapolation Versus Simulation

Knowing the number of protons translocated per electron during electron-transport (H^+^/e^–^ ratio) is of fundamental importance for chemiosmotic considerations. It is impossible to measure all translocated protons upon addition of a small electron acceptor pulse because the backflow via the membrane ATPase starts immediately while there are still protons being pumped out. Therefore, the classical way to calculate the H^+^/e^–^ ratio is based on a later phase of the pH curve, when there is no more electron transport going on. The backflow rate is assumed to follow first-order kinetics and to depend on the deviation from the initial transmembrane proton gradient, only. To calculate the number of translocated protons, a semi-logarithmic plot of the pH curve is made, the linear part of which is extrapolated to the time of electron acceptor addition.

Simulation by *proton.exe* also applies first order kinetics for proton backflow, but instead of a simple extrapolation to the time of acceptor addition it describes the different processes (uptake, reduction, and oxidation) by means of *K*_M_ and *V*_max_ values. This normally gives lower H^+^/e^+^ ratios, which, however, are more realistic. It appears obvious that the proton pumps cannot work at maximum rate at time zero, when the preceding steps (transport, sulfate activation etc.) are limiting factors. Furthermore, the simulation can easily mirror scalar pH effects that go on concomitantly.

### Steady-State Phases Are Typical for Reductant Pulse Experiments

Although in reductant pulse experiments the same metabolites are offered as in oxidant pulse assays, the physiological state of the cells is quite different. Here we have excess electron acceptors, and a more positive redox potential due to N_2_ instead of H_2_ saturation of the cell suspensions. During the pre-incubation phase the cells will run into depletion of internal electron donors and form oxidized cytoplasmic metabolites, which were reduced instead of sulfate or nitrate when H_2_ became available. This explains why in reductant pulse experiments we did not find the scalar pH effects predicted by the Eqs 1–4.

We observed electron-transport driven proton translocation with different kinetics under these conditions. The rates were lower and there were steady-state phases of equal proton export- and backflow rates, particularly after increasing H_2_ additions. Extrapolation of the final phase of proton backflow to calculate the H^+^/e^–^ ratio is not adequate under these conditions and will give too high values. Simulation of the involved processes, however, is an appropriate approach for the assessment of experiments with steady-state phases.

### Using *proton.exe* to Understand Chemiosmotic Processes

By means of *proton.exe* not only pH changes and bulk metabolite concentrations are calculated. The simulation also indicates the amounts of conserved ATP with adjustable H^+^/ATP ratios as well as changes of the proton-motive force and its components. Trying out simulations with varied parameters (see Supplementary Material for the simulation parameters) shows which parameters are limiting the overall reaction rates and provide a deeper understanding of chemiosmotic principles.

Although there are several variables used in *proton.exe*, the search for the best simulation parameters will lead to similar results, particularly with respect to the limiting factors. We could verify the quality of our simulations by doing experiments with sequentially increasing pulses, thereby challenging the applied kinetic parameters. Variation of non-limiting factors hardly changes the simulation outcome, e.g., if for an oxidant pulse experiment the excess electron donor concentration is varied by a factor of 10 or even 100. Simulation thus unravels the relevant factors (The following examples are explained in the tutorial videos on www.microbial-world.com/proton.htm).

#### Example: ATPase Rate

It is impossible to simulate the pH curves of oxidant pulse experiments (in the absence of KSCN) if one assumes that there is electron-transport driven proton translocation and an ATPase rate below 0.5 s^–1^ (meaning that half of the translocated protons are flowing back within a second). Otherwise simulated curves will start to look like those obtained with KSCN. However, if one plots the membrane potential as well, it turns out that the ATPase rates must be much closer to 1.0. Even with a value of 0.95 s^–1^, the simulated membrane potential transiently increases to values beyond −600 mV (e.g., simulation of sulfate reduction in Figure 1). The latter value is very unrealistic and would make the membrane leaky as shown by Kirchhoff and Cypionka (2017). With an ATPase rate of 0.99 s^–1^ the membrane potential still changes transiently by −80 mV. In experiments with KSCN, that neutralizes the membrane potential, the ATPase rates in our simulations decreased drastically to values below 0.1 s^–1^, showing that the membrane potential, and not ΔpH is the major player in chemiosmotic energy conservation.

#### Example: Cytoplasmic Buffering Capacity

Another simulation result shows the strong buffering capacity of the cytoplasm. Based on unpublished experiments, we assumed in our simulations a buffering capacity of the cytoplasm (1 mV/mM H^+^ added) ten times lower than that reported for bacilli and *Escherichia* coli by Krulwich et al. (1985). However, even if with that assumption and with an ATPase rate of 0.95 s^–1^ (giving the strong response of the membrane potential described above) the cytoplasmic pH changes transiently by only 0.02 units (≅1 mV). This is quite astonishing as a bacterial cell with an cytoplasmic pH of 7.7 harbors only about 5 free H^+^ (to calculate this consider that pH 8 means 10^–8^ mol H^+^ per liter and that the cytoplasmic volume is below 10^–15^ liter, so there will be 10^–23^ mol or 6 free H^+^ in a large bacterium). However, amino acids and nucleic acids effectively buffer the cytoplasm, whereas the membrane potential – although generated by >30,000 charges per cell – is much more sensitive to electrogenic processes.

#### Example: Electroneutral or Electrogenic Proton-Sulfate Symport

The last example shows why electrogenic proton symport (for example with sulfate) remains invisible to the pH electrode. At non-limiting concentration, sulfate uptake is electroneutral with 2 H^+^ per ${\text{SO}}_{4}^{2-}$ (Cypionka, 1995), and disappearance of two protons per sulfate can be measured with the pH electrode. During growth under sulfate limitation *D. desulfuricans* is known to induce an electrogenic proton-sulfate symport mechanism with 3 H^+^ per ${\text{SO}}_{4}^{2-}$ (Cypionka, 1989, 1994). However, simulation of the two different situations (electroneutrally with 2 H^+^ per electron acceptor, or electrogenically with 3 H^+^, with the third H^+^ lowering the membrane potential by +1 charge) do not show any differences in the pH curves. This can be explained by the extremely fast backflow via the ATPase rate as described above. The only visible change in the simulation is a reduced ATP gain after electrogenic uptake, as one proton less contributes to ATP conservation.

## Conclusion

Recording and simulating proton-related processes is a powerful approach to assess chemiosmotic energy metabolism at micromolar concentrations and time scales of seconds to minutes. The simulation app *proton.exe* is unique in relating pH curves (1) to biochemical reactions consuming or producing protons, (2) to proton-anion symport, and (3) to electron-transport-driven vectorial proton translocation. The simulation describes the kinetics of the reactions (*K*_M_ and *V*_max_) and displays possible ATP gains and changes of the transmembrane pH gradient and electrical membrane potential. In our study we demonstrated its use with a sulfate-reducing bacterium as model organism. However, comparable simulations are applicable to all bacteria or mitochondria and easily adaptable to light-driven proton translocation by photosynthetic bacteria or chloroplasts.

## Data Availability Statement

The raw data supporting the conclusion of this article will be made available by the authors, without undue reservation.

## Author Contributions

J-OR performed the experimental work, did the simulations, and wrote a draft version of the manuscript. HC designed the study, wrote the software, and wrote the final version of the manuscript. Both authors contributed to the article and approved the submitted version.

## Conflict of Interest

The authors declare that the research was conducted in the absence of any commercial or financial relationships that could be construed as a potential conflict of interest.
