# Supplementary material for: Recording and Simulating Proton-Related Metabolism in Bacterial Cell Suspensions
Source: Front Microbiol. 2021 Apr 29;12:654065. doi: 10.3389/fmicb.2021.654065 (PMC8117226; doi:10.3389/fmicb.2021.654065)
Supplement: Supplementary file 2 [file Data_Sheet_2.PDF]

[] General

Manual of

P R O T O N . E X E

(c) Heribert Cypionka, Univ. Oldenburg

Version: 2021-03-03

PROTON.EXE is a portable freeware app that simulates metabolic processes in order to explain pH effects one might find in cell suspensions. This includes uptake and reduction of an electron acceptor, oxidation of an electron donor, and the electron transport-coupled proton translocation. At the same time changes of the membrane potential, ΔpH and ATP conservation are simulated.

[] Installation

Download the newest version (proton.zip) from

www.microbial-world.com/freewarelist.htm

There is no installation required, just unzip the file 'proton.exe'. Proton does not change any system parameters.

[] Decimal separator

For correct processing of floating point numbers it is recommended to set the point as decimal separator under 'Regional Settings'.

[] Removal

There is no deinstallation required, just delete the file 'proton.exe'.

[] Freeware

Proton is a test program. It is freeware that you use on your own risk.

The author does not guarantee that it is working correctly and will not take any responsibility for any damage users might experience by using it.

[] Open a file

Click on File -> Open and select a Proton parameter File (*.H+). Only files following exactly the structure decribed below will be read correctly.

[] File format of H+ files

The parameters are saved in a text file that can also be loaded as CSV file with Excel or compatible programs. The semicolon ";" is used as separation character.

The data format is:

Headline 1: Copyright
Headline 2: Experiment description (= headline above data table)
Next 61 lines: Parameter number (01 ... 61): Parameter name ; Parameter value

Data: Copy of the calculated data panel shown on the right side

File Example:

```
H+ Proton Parameter and Data File, (c)1986-2021 Heribert
Cypionka
Description: Demo
 1: Cell suspension OD ;  10.0000
 2: Cells per ml (OD=1) [*10^8] ;  3.2800
 3: Volume/cell [*10^-15 l] ;  0.3870
...
61: Active Channels (Code 1+2+4+8) ;  15.0
(Order, name and units of listed data:)
Time (s); pH (mV); Acc.(out); Acc.(red); ATP cons.; MP(mV);
pHi; pmf (mV)
(Calculated data:)
  0;-9,30;990,00;0,00;0,00;-130;7,70;-162
  2;-9,30;970,00;0,00;0,00;-130;7,70;-162
...
(Number of lines depends on the steps that have been
calculated accoto 'Step' and 'Stop after (sec)')
```

[] Save Data

Lets you select a folder and file name (*.H+) to save your parameters in the format described above. Instead of '.H+' you could also name your file with '.txt' or '.csv'. The files can be read by text editors or spread sheet programs

(such as Microsoft Excel).

[] Exit Program

Quits without checking whether data are saved.

[] Options

- Allows for omitting symbols and grid in the graphical plot.

- Allows for plotting membrane potential and pH(i) instead of electron acceptor and reduced acceptor

[] Cell volume and surface

If one assumes the cell to be a rod with rounded ends one can describe it as a cylinder with two hemispherical caps at the ends. Then the following parameters to calulate cell volume and surface can be applied:

```
Assume: Cell length   len = 1.5 µm
        Cell width    wid = 0.5 µm
                      pi = 3.14

Radius                r = wid/2 = 0.5 µm/2 = 0.25 µm
Circular area         a = pi r^2 = 3.14 * 0.25^2 = 0.196
µm^2
Circumference         cir := 2 pi * r = 2* 3.14 * 0.25 =
1.57 µm

Cylinder height       cylh = len -2r =1.5- 2*0.25 = 1 µm
Cylinder mantle area  cyla = cir * cylh = 1.57 µm^2
Cylinder volume       cylvol = cylh * a  = 1*0.196 =0.196
µm^3
```

The two caps add to a sphere, therefore:

```
Cap area              capa = 4 * pi * r^2 = 4* 3.14 * 0.25^2
= 0.785 µm^2
Cap volume            capvol = (4/3) * pi * r^3 = 1.333*
3.14 * 0.25^3 = 0.785 = 0,0654 µm^3
```

For the complete cell follows:

```
Cell volume           cellvol = cylvol + capvol = 0.196 +
0.065 = 0.261 µm^3
Cell surface          cella =  cyla + capa = 1.57 + 0.785
µm^2
                             = 0.785 * 10^-8 cm^2
```

[] Membrane capacity

Based on Hodgkin & Nakajima (1972) J. Physiol. 221:121-136

Constant: 1 µF/cm^2 = 1.036*10^-11 mol or 6.24*10^12 e- per V and cm^2

F = 96.5 C/mol = 96 500
1 farad = 1 coulomb / 1 volt
1 coulomb = 1 A * s

[] Calculate data

- proton.exe calculates results once per second. If 'Step' is set to values >1 sec, the calculations are unchanged, but only part of the results are shown.

- The amounts added are assumed to be nmol. This corresponds to µmol per Liter or µM for a reaction volume of 1 ml.

- Transport and biochemical reactions rates (v) are described by a maximum rate (vmax, nmol per sec), a Km value (concentration at half maximum rate) and the current substrate concentration [S] according to

```
     vmax * [S]
v = -----------
      [S] + Km
```

For intracellular processes, Km and [S] refer to the cytoplasmic volume.

- Responses of the pH electrode and the ATPase rate (both 1/sec)
are assumed to follow first-order kinetics.

- For the pH electrode the values depend on the difference to the final (real) mV signal according to:

displayed signal = current signal + R*(final value - current signal)

with R = Response of the pH electrode per sec (0...1)

The current ATPase activity is assumed to be proportional to the
difference from the initial transmembrane pH gradient (t=0, which
is assumed to be in equilibrium) according to:

H+ uptake = A . (ΔpH - ΔpH0)

with A = ATPase H+ uptake rate per sec (0...1)
and ΔpH resp. ΔpH0 being the current or initial proton gradient. (The membrane potential is not considered as it is assumed to be neutralised chemically.)

- Some additional data (electron acceptor and donor amount, ATP conserved, membrane potential and the cytoplasmic pHi) are calculated as well.

[] Graphics

There is a simple graphical plot (with adjustable scales) showing the simulated pH curve and (optionally) changes of the electron acceptor, reduced acceptor and ATP concentrations. Instead of the oxidised and reduced electron acceptor concentrations, display of membrane potential and ΔpH can be selected under 'Options'. For a more sophisticated display please save the simulation as H+ (or csv) file and read data with a spreadsheet program.

[] Tutorial

Several tutorial videos are available under
www.microbial-world.com/proton.htm

[] Finally

Please be patient when playing with proton.exe. Chemiosmotic processes are not always easy to understand, but they are among the the most common processes in every living cell. It took me more than 30 years to design the current simulation. Just contact me, if you have questions or ideas how to improve it.

Good luck and enjoy!

Heribert Cypionka

Heribert.Cypionka@uni-oldenburg.de

www.microbial-world.com
