# Supplementary material for: Recording and Simulating Proton-Related Metabolism in Bacterial Cell Suspensions
Source: Front Microbiol. 2021 Apr 29;12:654065. doi: 10.3389/fmicb.2021.654065 (PMC8117226; doi:10.3389/fmicb.2021.654065)
Supplement: Supplementary file 4 [file Table_1.pdf]

Supplement Table S1

Standard deviations between measured and simulated data points

Figure	Addition	St dev. %
**2**	Sulfate & Nitrate	2.65
**3**	Sulfate	3.99
**4**	Nitrate	4.63
	Nitrite	2.78
**5A**	H_2 (68 nmol)	6.81
	H_2 (34 nmol)	14.1
	H_2 (13.6 nmol)	16.6
**5B**	H_2 (68 nmol)	7.28
	H_2 (34 nmol)	7.30
	H_2 (13.6 nmol)	13.2
